# Supplementary material for: The Analysis of Human Serum N-Glycosylation in Patients with Primary and Metastatic Brain Tumors
Source: Life (Basel). 2021 Jan 6;11(1):29. doi: 10.3390/life11010029 (PMC7825111; doi:10.3390/life11010029)
Supplement: Supplementary file 1 [file life-11-00029-s001.pdf]

The Alterations of Human Serum N-glycome in Patients with Primary and Metastatic Brain Tumors

Supplementary Information

Csaba Váradi [1,*], Viktória Hajdu [1], Flóra Farkas [2], Ibolya Gilányi [2], Csaba Oláh [2] and Béla Viskolcz [1]

[1] Institute of Chemistry, Faculty of Materials Science and Engineering, University of Miskolc, 3515 Miskolc, Hungary; kemviki@uni-miskolc.hu (V.H.); bela.viskolcz@uni-miskolc.hu (B.V.)
[2] Borsod-Abaúj-Zemplén County Center Hospital and University Teaching Hospital, 3526 Miskolc, Hungary; vfflora@bazmkorhaz.hu (F.F.); gilanyi.lab@bazmkorhaz.hu (I.G.); olah.idegseb@bazmkorhaz.hu (C.O.)
* Correspondence: kemcsv@uni-miskolc.hu; Tel.: +36-30-8947730

Supplementary Figure 1. Enhanced fluorescent signal intensity of procainamide labeled dextran ladder compared to 2-AB and 2-AA

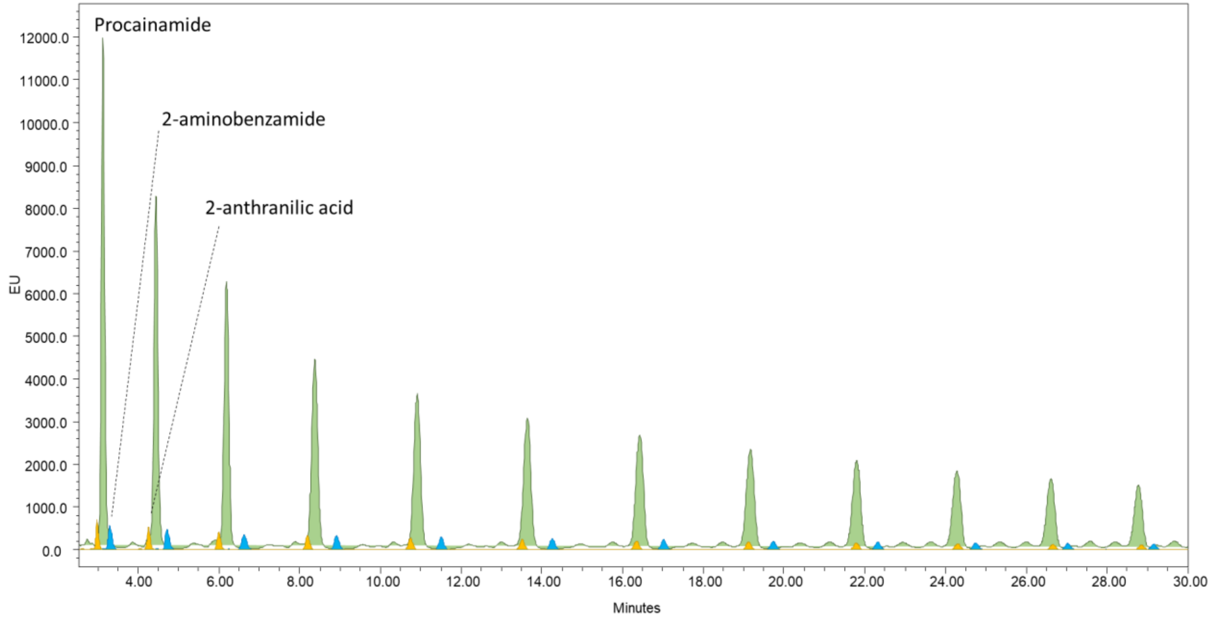

Supplementary Figure 2. Ionisation efficiency differences of procainamide, 2-AB and 2-AA labeled dextran ladders in positive MS mode

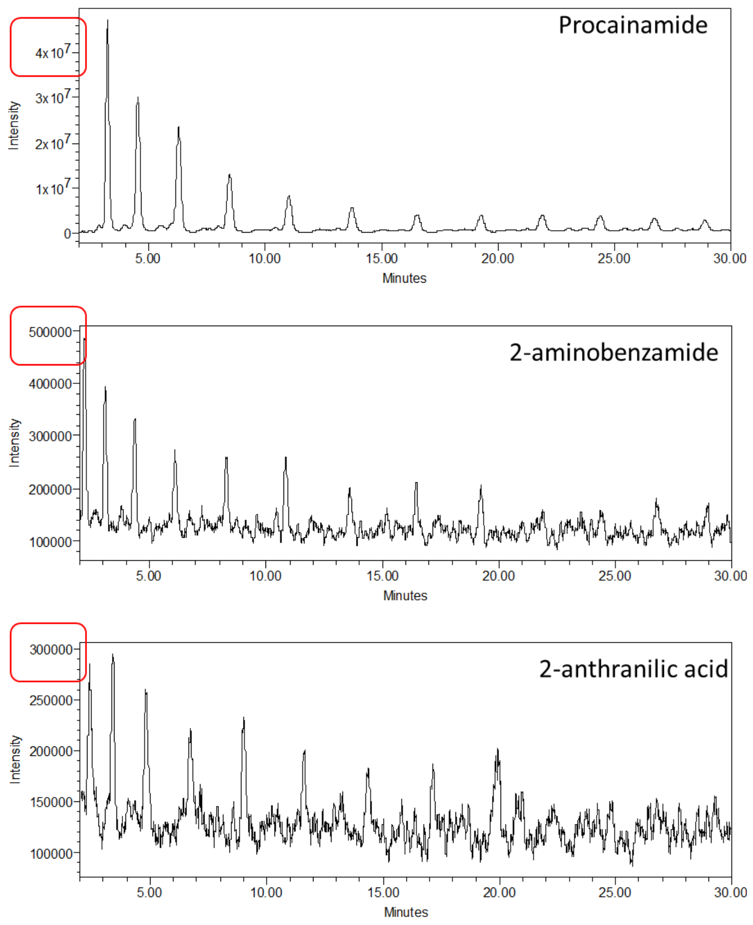

Supplementary Figure 3. Fluorescence and MS total ion chromatogram profile of sialidase digested serum N-glycome

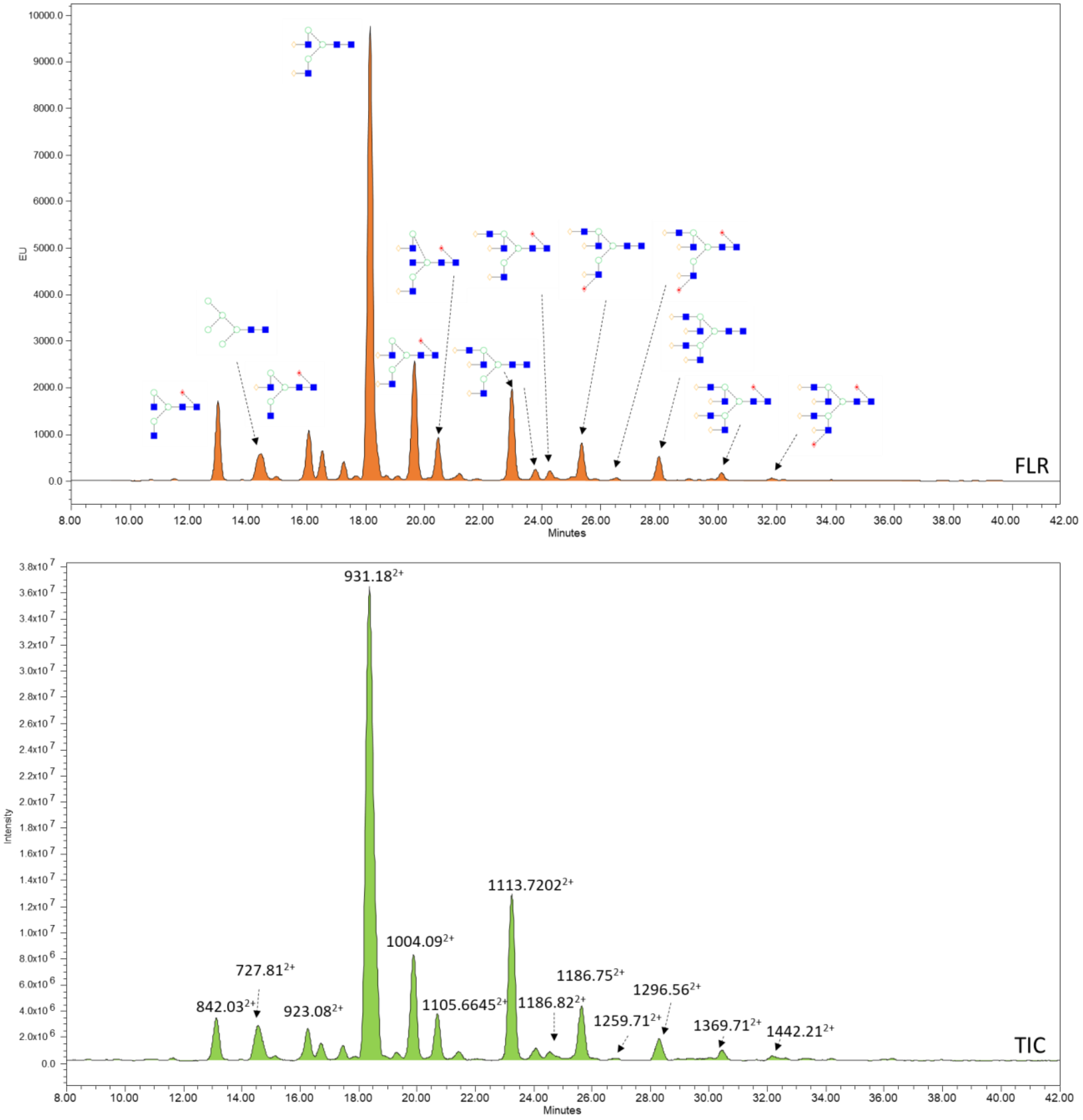

Supplementary Figure 4: Linkage-specific fucosidase digestions of sialidase treated serum N-glycans to identify α1-6 and α1-3/4 linked fucose residues

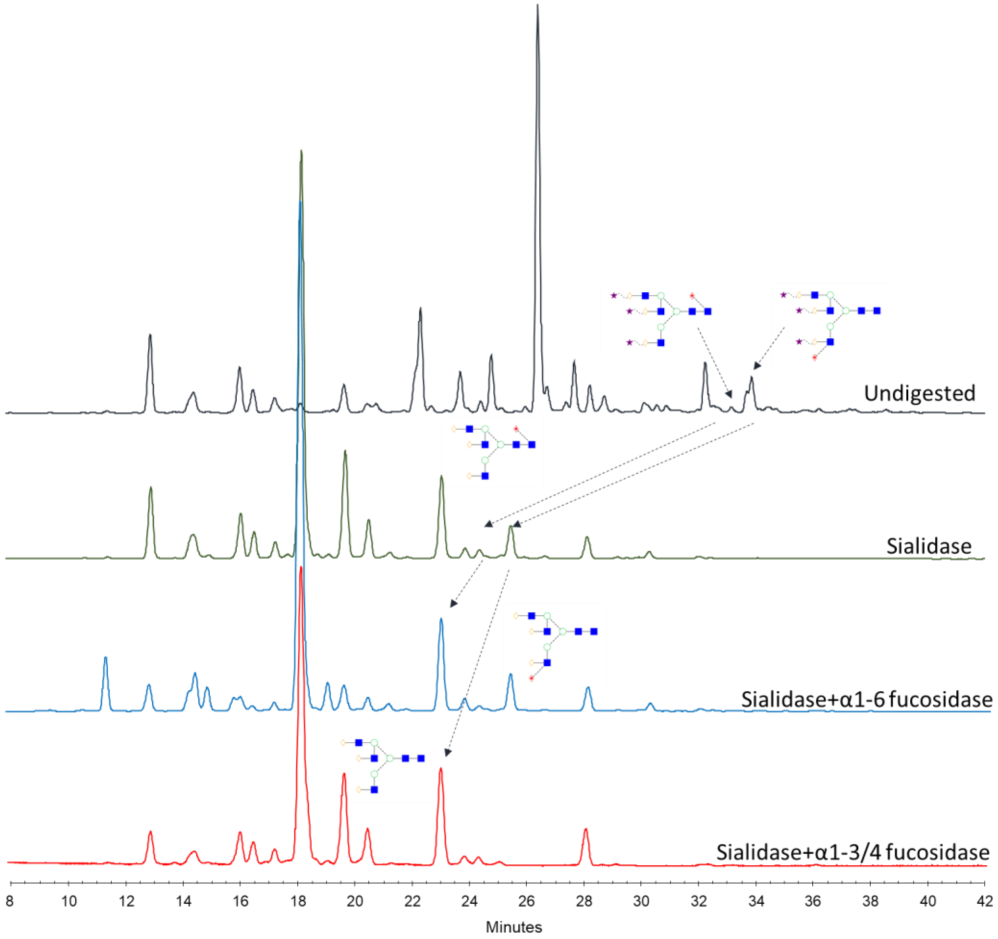

Supplementary Figure 5: The main tendencies comparing healthy controls to the brain cancer patients were the higher level of bi-antennary neutral and lower level of highly sialylated structures

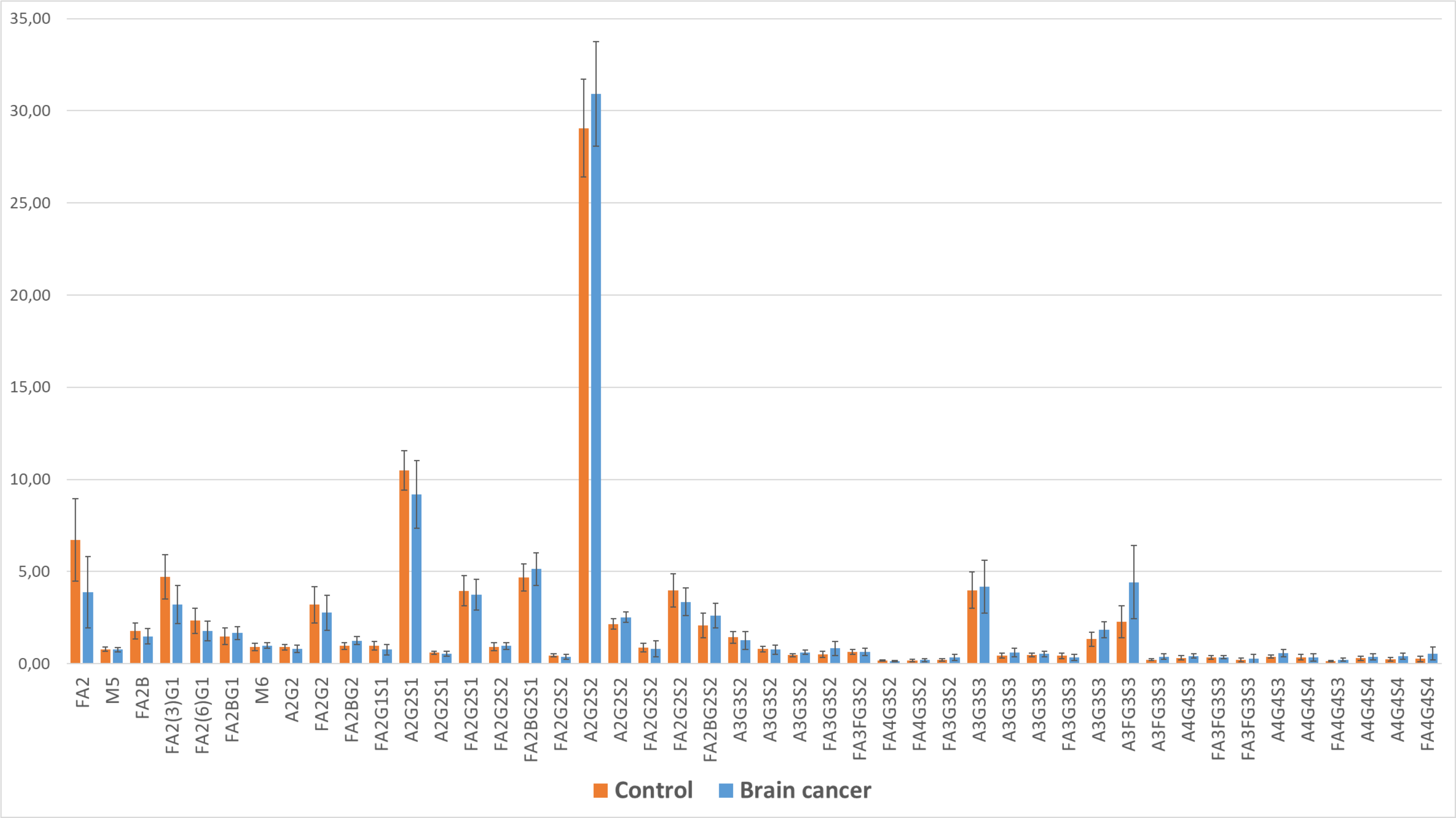

Supplementary Table 1: Quantified serum N-glycans in healthy controls and patients with different brain tumors (meningioma, glioblastoma, metastasis)

						Control		Meningeoma		Glioblastoma		Metastasis		
	**Retention Time**	**Structure**	**Experimental m/z**	**Theoretical m/z**	**ppm**	**Area%_Mean**	***Std. Deviation***	**Area%_Mean**	***Std. Deviation***	**Area%_Mean**	***Std. Deviation***	**Area%_Mean**	***Std. Deviation***	**Significance (p value)**
Peak1	12.76	FA2	842.03	841.94	106.30	6.71	*2.23*	4.72	*1.82*	5.00	*1.73*	5.72	*2.34*	0.02
Peak2	14.15	M5	727.81	727.88	-96.58	0.79	*0.11*	0.72	*0.12*	0.73	*0.10*	0.63	*0.13*	0.01
Peak3	14.25	FA2B	943.57	943.48	92.53	1.77	*0.44*	1.18	*0.28*	1.30	*0.44*	1.33	*0.50*	0.00
Peak4	15.83	FA2(3)G1	923.10	922.97	144.75	4.71	*1.19*	2.91	*1.13*	3.42	*1.30*	3.16	*0.67*	0.00
Peak5	16.29	FA2(6)G1	923.06	922.97	101.41	2.33	*0.69*	1.38	*0.46*	1.71	*0.73*	1.62	*0.34*	0.00
Peak6	17.04	FA2BG1	1024.57	1024.51	60.22	1.49	*0.44*	0.88	*0.28*	0.99	*0.42*	1.05	*0.37*	0.00
Peak7	17.63	M6	808.86	808.91	-57.24	0.90	*0.19*	0.70	*0.15*	0.78	*0.13*	0.74	*0.16*	0.00
Peak8	17.91	A2G2	931.07	930.96	117.41	0.89	*0.14*	0.83	*0.22*	0.89	*0.16*	0.73	*0.18*	0.06
Peak9	19.42	FA2G2	1004.09	1003.99	97.01	3.20	*0.99*	1.70	*0.66*	2.26	*1.38*	1.89	*0.68*	0.00
Peak10	20.23	FA2BG2	1105.35	1105.53	-165.08	0.96	*0.19*	0.71	*0.16*	0.78	*0.29*	0.80	*0.21*	0.00
Peak11	20.58	FA2G1S1	1068.62	1068.51	99.11	0.99	*0.23*	0.79	*0.34*	0.77	*0.30*	0.86	*0.19*	0.03
Peak12	22.10	A2G2S1	1076.63	1076.51	110.26	10.49	*1.08*	12.49	*2.09*	11.47	*1.21*	10.02	*1.40*	0.00
Peak13	22.48	A2G2S1	1076.53	1076.51	18.49	0.60	*0.09*	0.75	*0.15*	0.67	*0.10*	0.57	*0.09*	0.00
Peak14	23.48	FA2G2S1	1149.45	1149.54	-77.68	3.96	*0.81*	3.33	*1.07*	3.06	*0.87*	3.19	*0.53*	0.00
Peak15	24.22	FA2G2S2	1295.23	1295.09	106.63	0.92	*0.21*	0.74	*0.19*	0.68	*0.16*	0.66	*0.19*	0.00
Peak16	24.58	FA2BG2S1	1251.15	1251.08	55.79	4.69	*0.74*	4.67	*0.98*	4.85	*1.02*	4.79	*0.73*	0.98
Peak17	25.77	FA2G2S2	1295.30	1295.09	162.46	0.45	*0.08*	0.42	*0.09*	0.44	*0.19*	0.37	*0.11*	0.08
Peak18	26.21	A2G2S2	1222.24	1222.06	150.89	29.05	*2.65*	33.23	*3.21*	32.31	*2.47*	31.22	*2.83*	0.00
Peak19	26.52	A2G2S2	1222.25	1222.06	157.28	2.16	*0.27*	2.39	*0.30*	2.35	*0.17*	2.27	*0.36*	0.04
Peak20	27.18	FA2G2S2	1295.22	1295.09	101.77	0.88	*0.25*	1.03	*0.33*	1.40	*0.30*	1.61	*0.46*	0.00
Peak21	27.46	FA2G2S2	1295.20	1295.09	84.86	3.98	*0.91*	3.74	*0.79*	3.69	*0.83*	3.81	*0.74*	0.82
Peak22	28.02	FA2BG2S2	1396.42	1396.63	-148.86	2.08	*0.66*	1.79	*0.49*	1.39	*0.43*	2.48	*0.56*	0.00
Peak23	28.51	A3G3S2	1404.42	1404.63	-144.66	1.43	*0.31*	1.76	*0.60*	1.57	*0.34*	1.26	*0.39*	0.10
Peak24	29.90	A3G3S2	1404.44	1404.63	-130.57	0.80	*0.15*	0.89	*0.31*	0.69	*0.14*	0.60	*0.20*	0.02
Peak25	29.98	A3G3S2	1404.63	1404.63	5.84	0.47	*0.08*	0.54	*0.11*	0.44	*0.14*	0.46	*0.12*	0.15
Peak26	30.33	FA3G3S2	1477.45	1477.65	-140.63	0.50	*0.17*	0.64	*0.31*	0.75	*0.43*	1.00	*0.33*	0.00

Peak27	30.69	FA3FG3S2	1550.60	1550.68	-50.88	0.63	*0.14*	0.72	*0.22*	0.72	*0.19*	0.64	*0.21*	0.46
Peak28	30.97	FA4G3S2	1053.19	1053.13	53.74	0.16	*0.04*	0.18	*0.06*	0.15	*0.03*	0.14	*0.03*	0.16
Peak29	31.15	FA4G3S2	1053.17	1053.13	37.89	0.17	*0.07*	0.19	*0.06*	0.24	*0.07*	0.24	*0.09*	0.01
Peak30	31.75	FA3G3S2	1477.73	1477.65	51.23	0.21	*0.07*	0.25	*0.14*	0.27	*0.16*	0.40	*0.12*	0.00
Peak31	32.04	A3G3S3	1033.65	1033.78	-130.10	3.99	*0.98*	4.45	*1.76*	4.14	*1.25*	3.39	*1.22*	0.44
Peak32	32.32	A3G3S3	1033.69	1033.78	-95.67	0.43	*0.13*	0.48	*0.20*	0.54	*0.26*	0.60	*0.21*	0.07
Peak33	32.46	A3G3S3	1033.88	1033.78	92.38	0.46	*0.11*	0.56	*0.18*	0.51	*0.17*	0.51	*0.14*	0.22
Peak34	32.95	FA3G3S3	1082.53	1082.47	55.89	0.42	*0.14*	0.43	*0.15*	0.50	*0.18*	0.44	*0.18*	0.66
Peak35	33.51	A3G3S3	1033.65	1033.78	-130.49	1.33	*0.40*	1.57	*0.48*	1.36	*0.46*	1.38	*0.39*	0.55
Peak36	33.65	A3FG3S3	1082.41	1082.47	-57.74	2.27	*0.86*	2.83	*1.72*	3.39	*1.97*	5.10	*1.76*	0.00
Peak37	33.90	A3FG3S3	1082.50	1082.47	27.71	0.21	*0.05*	0.26	*0.14*	0.28	*0.13*	0.39	*0.15*	0.00
Peak38	34.17	A4G4S3	1155.45	1155.50	-40.85	0.32	*0.11*	0.42	*0.13*	0.42	*0.14*	0.36	*0.09*	0.02
Peak39	34.26	FA3FG3S3	1131.21	1131.16	46.77	0.35	*0.10*	0.38	*0.09*	0.37	*0.11*	0.39	*0.08*	0.30
Peak40	34.48	FA3FG3S3	1131.30	1131.16	126.95	0.21	*0.10*	0.25	*0.16*	0.33	*0.18*	0.58	*0.29*	0.00
Peak41	35.47	A4G4S3	1155.58	1155.50	69.49	0.39	*0.09*	0.48	*0.21*	0.50	*0.22*	0.56	*0.14*	0.01
Peak42	36.00	A4G4S4	1252.58	1252.53	45.03	0.34	*0.15*	0.40	*0.21*	0.47	*0.21*	0.38	*0.17*	0.22
Peak43	36.87	FA4G4S3	1204.06	1204.18	-102.31	0.13	*0.04*	0.15	*0.11*	0.21	*0.10*	0.21	*0.07*	0.01
Peak44	37.07	A4G4S4	1252.66	1252.53	106.19	0.29	*0.11*	0.36	*0.18*	0.39	*0.20*	0.35	*0.15*	0.07
Peak45	37.25	A4G4S4	1252.69	1252.53	127.50	0.25	*0.09*	0.29	*0.17*	0.35	*0.17*	0.42	*0.19*	0.01
Peak46	38.35	FA4G4S4	1301.12	1301.21	-68.01	0.26	*0.14*	0.41	*0.32*	0.48	*0.29*	0.72	*0.38*	0.00
